# *ceh-84,* an unusual homeobox gene

**DOI:** 10.17912/micropub.biology.000340

**Published:** 2020-12-01

**Authors:** Molly Reilly, Oliver Hobert

**Affiliations:** 1 Columbia University, Department of Biological Sciences; 2 Howard Hughes Medical Institute

**Figure 1:**
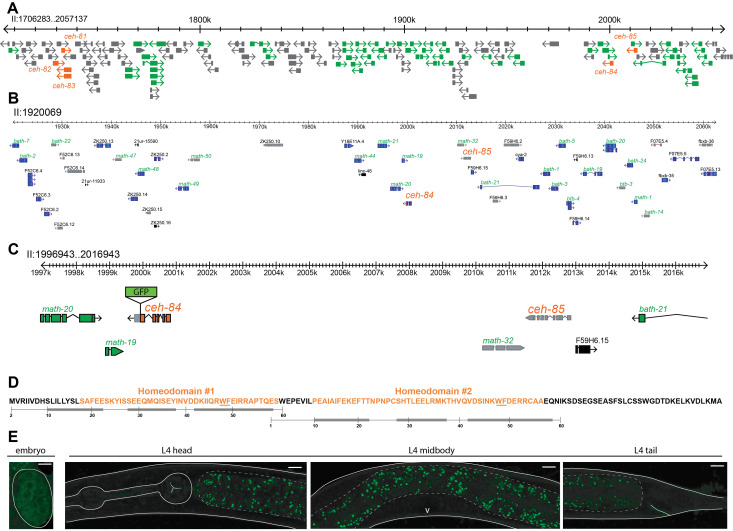
Location, features and expression of the *ceh-84* homeobox gene. A: Parts of the previously described *C. elegans­*-specific gene cluster on chromosome II are shown. The homeobox genes *ceh-81* through *ceh-85* are outlined and labelled in orange and are surrounded by MATH and BTB type genes in green. All other genes and non-coding RNAs are colored gray. B: *ceh-84* and *ceh-85* genomic region with numerous pseudogenes highlighted in gray, protein coding genes in blue, and non-coding RNAs in black. MATH and BTB type genes labelled in green, homeobox genes labelled in orange, all others in black. C: Further zoom in on *ceh-84* and *ceh-85* loci showing schematic of *ceh-84* reporter with insertion of GFP sequence before the stop codon. Pseudogenes are colored in gray, protein coding genes belonging to the homeobox family in orange, protein coding genes belonging to the MATH and BTB type in green, all others in black. D: CEH-84 contains two unusual, short homeodomains. Orange indicates the sequence match to the SSF46689 superfamily domain, a homeodomain signature automatically generated by http://supfam.org. The numbering below indicates the position of canonical, usually 60 aa long homeodomains and their predicted alpha helices (gray bars), with helix 3 binding in the major groove of DNA (Bürglin and Affolter, 2016). This numbering is anchored with the highly conserved WF residues (underlined). E: Images of CEH-84 protein fusion reporter, PHX2811 (allele *syb2811*), generated by insertion of *gfp* sequence by CRISPR/Cas9. On the left, embryonic GFP expression looks diffuse and non-nuclear. On the right, no detectable GFP in L4 hermaphrodites. Solid white lines outline the worm structures, dashed lines highlight the intestinal GFP autofluorescence, and the white V marks the vulva. Using a ~2kb promoter fusion, postembryonic intestinal expression was observed by McKay *et al.*, 2003 and weak embryonic expression was noted by Hench at al., 2015.

## Description

We have previously described the expression patterns of all but one of the 102 homeobox genes present in the *C. elegans* genome, using either fosmid-based reporter transgenes or CRISPR/Cas9-engineered *gfp* reporter alleles (Reilly *et al.*, 2020). The last remaining homeobox gene for which we were initially not able to generate a reporter reagent is *ceh-84* (Reilly *et al.*, 2020). As previously recognized by Thomas Bürglin (Hench *et al.*, 2015), *ceh-84* codes for an unusual homeodomain protein with two divergent homeodomains (**Fig.1D**). It likely is a tandem duplicate of the *ceh-85* gene, which has been classified as a pseudogene (**Fig.1B**). *ceh-84* has no recognizable homologs in other *Caenorhabditis* species (Hench *et al.*, 2015). Together with its duplicate, *ceh-85*, it localizes to a large cluster of extensively duplicated genes on chromosome II, described by James Thomas (Thomas, 2006). Most of the duplicated genes code for MATH and BTB type proteins (**Fig.1A**). The entire cluster seems to be *C. elegans-*specific (Thomas, 2006) and, apart from a number of pseudogenes, it also contains the *C. elegans*-specific *ceh-81*, *ceh-82* and *ceh-83* homeobox genes (**Fig.1A**).

We designed a *gfp* reporter allele of *ceh-84* that was generated by CRISPR/Cas9 genome engineering. We observed no expression of the protein in any cells in larvae and adults (**Fig.1E**). In embryos we observe very dim, diffuse GFPsignals that make it unlikely that any functional protein is present in the nucleus at any stage. The absence of obvious CEH-84 in the mature nervous system is consistent with our previously reported observation that nematode or *C. elegans-*specific homeobox genes make no significant contribution to the combinatorial code of homeobox gene expression in the nervous system (Reilly *et al.*, 2020).

## Reagents

PHX2811 *– ceh-84(syb2811[ceh-84::GFP]).* Generated and provided by Sunybiotech.

## Methods

For imaging, worms were anesthetized using 100mM of sodium azide (NaN_3_) and mounted on 5% agarose pad on glass slides. Images were acquired at 40x using a confocal laser scanning microscope (Zeiss LSM880) and processed using the ImageJ software. Representative maximum intensity projections are shown for GFP channel as gray scale and gamma and histogram were adjusted for visibility.
